# Item development for a patient‐reported measure of compassionate healthcare in action

**DOI:** 10.1111/hex.13953

**Published:** 2024-01-21

**Authors:** Eleanor Chatburn, Elizabeth Marks, Lucy Maddox

**Affiliations:** ^1^ Psychology Department University of Bath Bath UK; ^2^ University of Exeter Exeter UK; ^3^ Present address: DClinPsy, Lecturer University of East Anglia Norwich UK

**Keywords:** care provision, compassion, measure development, patient‐reported measure

## Abstract

**Background:**

Compassionate care is a fundamental component of healthcare today; yet, many measures of compassionate care are subjective in focus and lack clarity around what compassionate care looks like in practice. Measures mostly relate to physical healthcare settings, neglecting mental healthcare. They also lack significant involvement of people with lived experience (PLE) of healthcare delivery in their development. This study aimed to begin the process of developing a new patient‐reported measure, one that captures the observable actions of compassionate care delivery or ‘compassionate healthcare in action’ by any healthcare professional working in any care setting. The study involves PLE of healthcare delivery, both patients and staff, throughout.

**Methods:**

A multistage mixed‐methods scale development process was followed. First, items were derived inductively from reflexive thematic analysis of patient and clinician interviews about what compassionate care meant to them (*n* = 8), with additional items derived deductively from a literature review of existing measures. Next, a panel of patient, clinician and researcher experts in compassionate care was recruited (Round 1: *n* = 33, Round 2: *n* = 29), who refined these items in a two‐round modified online Delphi process.

**Results:**

Consensus was reached on 21 items of compassionate care in action relating to six facets: understanding, communication, attention, action, emotional sensitivity and connection. These items will form the basis for further scale development.

**Conclusions:**

This item development work has laid the foundation of a potential new tool to systematically measure what compassionate healthcare in action looks like to patients. Further research is underway to produce a valid and reliable version of this proposed new measure. We have outlined these initial stages in detail in the hope of encouraging greater transparency and replicability in measure development, as well as emphasising the value of involving PLE throughout the process.

**Patient or Public Contribution:**

This study involved PLE of both physical and mental healthcare (as staff, patients and service users) throughout the development of the new measure, including initial project conceptualisation and participation in item generation and refinement stages.

## 1 BACKGROUND

### 1.1 Compassion in healthcare

Compassion is a key component of healthcare, named as a core value in healthcare charters,^1^, ^2^ medical codes of ethics^3^ and guidance on patient‐centred care.^4^, ^5^ There is good evidence that compassionate care is associated with improved patient outcomes, including quicker recovery times, reduced anxiety,^6^ reduced rates of hospital readmission,^7^ lower rates of postdischarge posttraumatic stress disorder following emergency department admission^8^ and improved patient satisfaction.^7^ Patients have even said that compassionate care can make the difference between living and dying.^9^

However, despite widespread recognition of compassion's importance, a lack of compassionate care can still occur, with potentially devastating consequences.^10^, ^11^ High‐profile investigations have identified multiple risk factors that can influence compassionate care provision, including resource shortages, high patient throughput, training issues, poor organisational culture, low staff morale, burnout and inadequate leadership.^10^, ^12^, ^13^, ^14^

Subsequently, recent years have witnessed a rapid increase in empirical studies of compassion in healthcare^15^, ^16^ as well as initiatives aimed at improving compassionate care, particularly in high‐income countries. Interventions range from organisation‐level programmes to embed compassionate leadership models^17^, ^18^ to educational, mentorship or therapeutic programmes for healthcare staff^19^, ^20^, ^21^, ^22^ (commentators have noted that fewer compassionate care initiatives have been undertaken in low‐ and middle‐income countries, which is a particular concern, given indications that healthcare workers in lower‐resourced health systems in these contexts may be particularly vulnerable to the effects of burnout and compassion fatigue^23^, ^24^). While these developments are encouraging, there have been some difficulties in establishing effectiveness, due to ways in which compassion is defined, measured and understood.^25^, ^26^ For healthcare services to achieve the World Health Organisation's ambition of developing ‘cultures of compassion’,^27^ clarity is needed on what the provision of compassionate care actually *is*.

### 1.2 Defining compassion

Compassion is widely defined as consisting of two components: ‘the feeling or emotion when a person is moved by the suffering for distress of another, and the desire to relieve it’.^28^ The motivational aspect differentiates compassion from the more passive construct of empathy; for while empathy is a feeling *with* the other, compassion is a feeling *for* the other^29^ crucially including a desire to help. The provision of compassionate care therefore requires competencies of (i) compassionate engagement (ability to notice suffering in others) and (ii) compassionate action (intention to help).^30^

### 1.3 Measuring patient reports of compassionate care

Capturing patient experience is a long‐established priority for the improvement of both physical^31^ and mental healthcare.^32^ Guidelines for patient‐reported tool development state that the active involvement of patients in all stages is essential.^33^, ^34^, ^35^ Appropriate levels of patient and service user involvement can improve the acceptability, relevance and quality of a new measure, just as insufficient involvement can undermine it.^36^, ^37^ Accordingly, the COSMIN guidelines recommend that the involvement of patients in item generation stages is particularly important for the content and face validity of a new measure.^33^

Despite these recommendations, many patient‐reported measures are primarily based on the views of staff with limited input from patients.^37^ One study that examined 189 patient‐reported outcome measures found that less than 7% of studies included patients in every stage of the development process and 26% of studies included no patient involvement at all.^35^

Patients are in a unique position to report on how they experience compassion in healthcare encounters. As Haslam^12^ ^,p.2^ states, ‘patients or relatives know when care is being delivered with compassion and when it is not’. Yet, within the compassion field, a landmark scoping review found that compassionate care studies ‘failed to adequately incorporate the understanding and experiences of patients’.^16^ ^,p.14^ Where the original review identified only two studies that explored patient perspectives, a recent update has found evidence of nine such studies.^15^ However, there remains a considerable imbalance in how compassion is understood and studied overall, with more studies reporting on clinician samples (27 papers) than patient samples (12 papers).^15^

Without attending to the voices of more patient groups, settings and contexts, it is possible that previous studies have overlooked some aspects of compassionate care that matter most to patients. Such limited patient involvement means that some researchers have questioned the specificity, clinical applicability and conceptual validity of existing studies.^16^ More research is needed to find a consensus about which aspects of the provision of compassion in practice matters most to patients.

### 1.4 Compassionate healthcare in action

While there has been increasing interest in how staff, patients or family members experience compassionate care (for review papers, see Malenfant et al.^15^, ^16^, ^38^), describing compassionate care during a healthcare encounter still requires closer attention. Multiple commentators have noted the value of identifying and describing the tangible behavioural aspects in healthcare encounters to inform the design of clinical educational programmes and further research.^16^, ^39^, ^40^ The nursing literature has a strong tradition of offering rich descriptions of the subtle ways in which professional caregivers can communicate their compassion for others, actions that can be hard to define, identify and measure in practice.^41^ Perry has described how nurses convey compassion practically through attending to the ‘essential ordinary’^42^; it is often the little things that seem to make a biggest difference to patients.^43^ Nonverbal expressions of compassion include facial expression, posture, tone of voice and use of touch.^38^, ^44^ Recent empirical work by Baguley et al.^45^ using topic modelling analysis, primarily with doctors, identified seven groups of behaviours that are considered compassionate physical healthcare encounters. It is less clear whether this same pattern of findings would be found in settings outside of primary care and from a wider range of health and care professionals, but these data provide a good foundation.

### 1.5 Evaluation of existing measures

Inspection of six existing patient‐reported measures of compassionate care (see Table 1) reveals a number of limitations.

**Table 1:** Existing patient‐reported measures of compassionate care identified through literature review.

Author (year)	Name of measure	Country	Definition of compassion used	No. of items (subscales)	Healthcare context	Scaling	Patient involvement in measure development?	Subjective or behavioural items?
Burnell and Agan (2013)^46^	Compassionate Care Assessment Tool	USA	‘A sympathetic consciousness of others' distress with a desire to alleviate it’. Consideration of ‘compassion in the spiritual context’.	28(4)	Acute hospitals. Measure refers to ‘the nurse’.	4‐point Likert scale from ‘not important’ to ‘very important’.	Item generation: existing measures. Item refinement: pilot study of 110 patients in one acute faith‐based hospital: asked to rate items on importance of each item to compassionate care. Selected top 28 rated items.	Majority subjective
Fogarty et al. (1999)^6^	Unnamed physician compassion scale	USA	‘A sympathetic concern for the suffering of another, together with the inclination to give aid or support or to show mercy’ (Webster dictionary).	5(0)	Female breast cancer patients. Measure refers to ‘the physician’.	Five pairs of words rated on a scale from 0 to 100.	Item generation and refinement: supported by previous qualitative research (no details given).	Mix of subjective and behavioural
Lown et al. (2015)^47^	Schwartz Center Compassionate Care Scale	USA	‘Compassionate care is when physicians, nurses and other caregivers recognise and validate the concerns, pain, distress or suffering of patients and their families and take action to address them’.	12(0)	Acute hospitals. Measure refers to ‘the doctor’.	10‐point Likert scale from ‘not at all successful’ to ‘very successful’.	Item generation: committee of 20 patients and carers developed initial criteria. Items refinement: five focus groups with patients, nurses, physicians.	Majority behavioural
Mercer et al. (2004)^48^	Consultation and relational empathy	UK	Relational empathy is: the ‘ability to (i) understand the patient's situation, perspective and feelings (and their attached meanings); (ii) to communicate that understanding and check its accuracy; and (iii) to act on that understanding with the patient in a helpful (therapeutic) way’.	10(0)	General medicine or primary care. Measure refers to ‘the doctor’ and ‘the consultation’.	5‐point Likert scale from ‘poor’ to ‘excellent’.	Item generation: ‘supported by our previous qualitative work on patient views’, but no specific information given. Items refinement: three pilot studies with patients from GP practices. Also piloted with 20 clinicians and 20 research experts.	Majority behavioural
Roberts et al. (2019)^49^	5‐Item tool to measure patient assessment of clinician compassion	USA	‘An emotional response to another's pain and suffering involving an authentic desire to help’.	5(0)	Hospital outpatient setting. Measure refers to ‘your provider’ (‘physicians, advanced nurse practitioners, physician assistants’).	4‐point Likert scale from ‘never’ to ‘always’.	Item generation: based on a literature review, then candidate items rated by four professional experts. Items refinement: pilot testing with 3325 patients from outpatient clinic.	All subjective
Sinclair et al. (2020, 2021)^50^, ^51^	Sinclair Compassion Questionnaire: long form (LF) and short form (SF)	Canada	‘A virtuous response that seeks to address the suffering and needs of a person through relational understanding and action’.	15(0) LF 5(0) SF	Participants living with life‐limiting illnesses in four care settings: acute care, hospice, long‐term care and homecare.	5‐point Likert scale from ‘strongly disagree’ to ‘strongly agree’.	Item generation: Based on previous studies including a patient model of compassion and qualitative studies. Literature review. Item refinement: Delphi process and cognitive interviewing.	Majority subjective

*Note*: ‘Patient involvement’ denotes the use of patients/service users/people with lived experience of compassionate care in the initial stages of the measure development process for each measure. Individual ratings of whether items had a ‘subjective’ or ‘behavioural’ focus, as rated by the research team, are shown in Supporting Information S1: Table A.

Abbreviation: GP, general practitioner.

First, few measures focus on capturing the specific, observable behaviours of professionals when they deliver care with compassion. Concrete behavioural descriptors represent the majority of items in only two measures. The consultation and relational empathy (CARE) measure^48^ itemises specific healthcare worker actions with clear descriptions of what these would look like (e.g., ‘Making a plan of action with you’), although there are some more ambiguously worded items (e.g., ‘Being positive’). Similarly, The Schwartz Center Compassionate Care Scale (SCCCS)^47^ includes a majority of behavioural items, mixed in with some more subjective or ambiguous items (e.g., ‘Gain your trust’).

In the other four measures, behavioural items delineating what compassionate actions look like are either inconsistent or lacking. Notably, all five items of the five‐item compassion measure^49^ ask patients to rate their subjective feelings about the experience of care versus actual observations (e.g., ‘How often do you *feel* your provider is considerate of your personal needs’) (emphasis added). The remaining three measures contain a mixture of items. The Sinclair Compassion Questionnaire (SCQ)^51^, ^52^ gives relatively more weight to subjective experience over observable behaviours (e.g., ‘My healthcare provider made me feel cared for’), as does the Compassionate Care Assessment Tool (CCAT)^46^ (e.g., ‘Excusing shortcomings’) and an unvalidated compassion scale^6^ (e.g., ‘Cares about the patient’).

Most existing measures are limited to care provision in a narrow range of physical health settings (especially primary care, emergency medicine, older adult or palliative care; see Table 1). Such measures may omit components of the patient experience essential to other settings. None of these existing measures appear to have been developed or validated in mental healthcare settings despite the well‐evidenced need to achieve parity of esteem between physical and mental healthcare^53^ and potentially different emphases relating to compassionate behaviours in mental and physical healthcare settings. Furthermore, existing studies tend to focus on care provided by a limited number of health professionals (primarily doctors or nurses; see Table 1). Patients usually encounter a wide variety of clinical and nonclinical healthcare staff (e.g., receptionists, cleaners), all of whom can provide aspects of compassionate care, and who contribute to the overall experience of receiving compassionate care within a service.

A further limitation of some of the existing measures are potential issues of conceptual clarity. Two measures are based on conceptual definitions that incorporate multiple theoretical constructs (e.g., empathy and compassion in the CARE Measure; compassion and spiritual needs in the CCAT). Another measure contains no reference to action‐orientated aspects of compassion care (five‐item compassion measure), and there are also some items in measures that could be considered circular (e.g., ‘Showing care and compassion’ in the CARE Measure).

Finally, the quality and extent of patient involvement in developing these patient‐reported measures vary (Table 1). Two measures did not report any patient involvement in the initial item generation phase. Another two referred to previously conducted qualitative work on patient experiences of compassionate care but did not provide specifics on how this related to item generation. The SCCCS and the SCQ both involved people with lived experience (PLE) of physical healthcare in item generation, although the latter was the only study to report the use of formal group consensus methods involving patients. None of the measures reported the involvement of PLE of mental healthcare.

In summary, despite numerous measures existing in this field, there is variable focus on the behavioural expressions of compassionate care, difficulty with breadth of clinical applicability, variability in conceptual clarity and often a lack of patient involvement. There is no one existing measure with a focus on compassionate healthcare in action that can be used across physical and mental health settings and that strongly incorporates patient involvement into its design. We suggest that this is a gap that a new measure of compassionate healthcare in action could usefully fill.

### 1.6 Aims

1. To conduct the initial development of items for a new patient‐reported measure with potential for broad clinical applicability to capture the observable behaviours or actions of compassionate care delivery by a healthcare professional working in any care setting.
2. To ensure the development of a robust tool by using clear conceptual definitions of compassionate healthcare, a clearly reported systematic measure development process and involvement of PLE of healthcare delivery (patients and staff) at all stages of the project.

## 2 METHODS

A multistage, mixed‐methods scale development process was followed using established guidelines^54^: (1) defining the construct of interest, (2a) item generation using qualitative interviews and (2b) literature review of existing measures and (3) item refinement using a modified online Delphi process. This is in line with best practice for item generation when developing scales in health research.^55^

### 2.1 Defining the construct of interest

The above‐cited definition of compassion framed the initial construct of compassionate care and guided the qualitative interview schedule and literature search terms. The initial project conceptualisation and design were informed by feedback from two clinician/researchers and one patient representative.

### 2.2 Item generation: Key informant interviews

#### 2.2.1 Participants

Eight people recruited via professional contacts and a university committee of PLE of physical and mental healthcare were interviewed. Inclusion criteria were intentionally broad: (1) personal expertise in compassionate care; (2) over 18 years of age; and (3) proficient spoken English. Personal expertise was defined as ‘significant personal interest in and experience of receiving compassionate care in any healthcare setting’ and was determined by self‐report. Examples of personal expertise included a long‐term user of mental health services, a person receiving intensive cancer treatment and a carer of a relative with a neurodegenerative condition. Purposive sampling ensured equal representation from clinicians, lay people, physical and mental healthcare. The sample was diverse in gender, age and healthcare area, although most were white European and from the Southern England (see Supporting Information S1: Table B). With no recommended sample size for key informant interviews in scale development,^56^ recommendations for sample size in reflexive thematic analysis (RTA) were followed.^57^

#### 2.2.2 Procedure

The design and reporting of this qualitative component were informed by the consolidated criteria for reporting qualitative research^58^ (see the Supporting Information material). The aim was to understand people's lived experiences of what compassionate care delivery looks like, to enable the creation of initial items and facets for a potential measure of compassionate care in action. Operating from the view that everyone will have been a user of the healthcare system at some point (as patient or relative), all participants were asked about personal experiences of receiving compassionate care with additional questions for clinicians on providing compassionate care. A semi‐structured interview schedule^59^ was developed by all three researchers through roundtable discussion (see Supporting Information S1: Table C). Demographics were collected. Interviews lasted 25 min on average. They were audio‐recorded and transcribed verbatim by the lead researcher (E. C.).

#### 2.2.3 Data analysis

Analysis of interview transcripts was informed by RTA.^57^, ^60^ RTA is a theoretically flexible method for ‘developing, analysing and interpreting patterns across a qualitative data set’.^57^ ^,p.4^ Thematic analysis has been used to explore service users' experiences of using health services and participating in treatments.^61^, ^62^ We used an inductive, bottom‐up, RTA approach to analyse the latent content of the data from the standpoint of a broadly critical–realist orientation. After an initial period of familiarisation, the main researcher manually highlighted recurring key words and concepts within each case, and these were grouped into initial semantic codes. Data analysis was conducted iteratively, and codes were revisited as more interviews were conducted. Initial codes were then grouped into initial themes, which were supported by illustrative quotations. The entire data set and initial themes were reviewed by a second member of the research team to ensure thematic coherence. All three researchers then manually mapped out a final list of generated themes (corresponding to potential candidate facets of the new measure) and subthemes (corresponding to potential candidate items for the new measure). Researcher reflexivity was maintained throughout data collection and analysis through ongoing team discussions about their personal and research experiences of compassionate care and related presuppositions.

### 2.3 Item generation: Literature review

#### 2.3.1 Procedure

The main researcher searched the PubMed database using the following search terms: ‘compassion’, ‘compassionate’, ‘tool’, ‘measure’, ‘questionnaire’, ‘scale’. Studies were included if patients rated their perception of the provision of compassion by any healthcare professional. Exclusion criteria included clinician‐rated or observer measures, measures not related to provision of compassion in a healthcare context and papers not published in English (scales that measured the provision of emotional care,^63^ the quality of patient–staff interactions,^64^ relational aspects of compassion^30^, ^65^ and clinician self‐report or observer measures^39^, ^40^ did not meet all the inclusion criteria). Existing reviews of compassion measures and compassion interventions in healthcare were hand‐searched for references. Experts in compassion research were approached and asked for recommendations of other measures. This process identified six (the CARE Measure was included as the authors' conceptual definition of ‘relational empathy’ incorporated both of Gilbert's sensitivity and action‐orientated components of compassion, and it has been used as a measure of patient‐perceived compassion in healthcare settings)^8^ existing patient‐reported measures of compassionate care (see Table 1).

#### 2.3.2 Data analysis

The resulting items were analysed and mapped against the themes and items proposed from the thematic analysis. Areas of discrepancy were highlighted, and discussion in the research team led to the addition of new items that were added to the table to produce an enhanced list of items (see Section 3).

### 2.4 Item refinement: Modified online Delphi process

#### 2.4.1 Participants

Participants recruited for a Delphi process constitute ‘a panel of informed individuals’.^66^ ^,p.1221^ In this study, expertise (and eligibility to participate) was defined as either significant research interest in compassion or in compassionate care, or significant clinical practice in the field of compassion or compassionate care, or significant personal interest in and experience of receiving compassionate care. Participants also self‐identified if their experience fell primarily within the categories of physical healthcare, mental healthcare, social care or a combination. With three overlapping subgroups (researchers, clinicians and lived experience) rather than one heterogenous group, a larger sample size was recruited.^67^ Delphi participants were recruited using purposive sampling^68^ via professional contacts, a PLE committee and social media (including Twitter callouts). Participant eligibility was assessed on the basis of self‐report, with eligibility checks by the lead researcher. Diversity characteristics of participants were monitored but the researchers did not actively recruit people with particular characteristics.

Most participants (see Supporting Information S1: Table D) self‐identified with more than one subgroup (e.g., as a researcher and a PLE). There was an equal distribution between patients and clinicians, but a smaller proportion of researchers. Good representation was achieved from across the different domains of healthcare, although mental healthcare was strongly favoured. The sample was primarily White British, and two‐thirds identified as female. The sample was fairly balanced in terms of age, although there were no participants under 25 years of age. Nearly all participants were UK residents and the majority lived in the south‐east or south‐west. Clinical professions represented included psychiatry, nursing, clinical psychology, physiotherapy and psychotherapy.

#### 2.4.2 Procedure

A modified online Delphi method with compassionate care experts was used to refine the list of candidate items and to establish initial construct validity.^54^ The Delphi technique is widely used in health services research and enables the views of a range of stakeholders to be combined into a final group consensus.^69^ Using close‐ended questionnaires that can be combined with space for participants to provide qualitative feedback on their ratings, a Delphi survey proceeds over multiple rounds until consensus is reached.^68^ No guidelines are established for the design, format or number of rounds of the Delphi process.^70^, ^71^ A traditional Delphi method starts with a qualitative first round, but this can be modified if the round one items are derived from a previous qualitative study or literature review.^68^, ^72^

Participants were emailed a link to an online survey (Qualtrics), where they accessed the participant information sheet and provided informed consent. Candidate items were presented, ordered by facet and participants rated each item (51 items in Round 1, 44 in Round 2) on a 9‐point Likert scale from 1 to 3 (‘item is not important to defining compassionate care’), 4–6 (‘item is important but not critical’) and 7–9 (‘item is critically important’). This scoring system was adapted from the GRADE guidelines^73^ and has been used in a number of Delphi studies.^72^, ^74^, ^76^

Participants could also provide qualitative feedback regarding the wording of a potential questionnaire name and introduction, individual item wording and theme classification.^68^ In Round 1, they could suggest additional items, an accepted modification to the Delphi technique.^70^

To mitigate attrition and retain a response rate above the recommended minimum of 70%,^77^ each round was open for 8 weeks, and nonrespondents or partial completers were sent up to four email reminders, including an option to leave the study. The survey was piloted twice, with minor layout changes as a result. The average completion time for all participants was 25 min for Round 1 and 11 min for Round 2.

Participants received feedback between rounds, including descriptive statistics, a summary of the qualitative feedback and an explanation of subsequent changes to the measure.^71^ Completers of Round 1 were emailed a link to the second round of the survey. The items and facets were organised in the same order as the previous round.

#### 2.4.3 Data analysis

Descriptive statistics were examined for demographics and response rates calculated per round. In line with Delphi methodology,^78^ consensus was defined a priori as the proportion of ratings within a predetermined range at the ≥75% agreement threshold. Following establishing scoring methods,^74^, ^75^ it was specified that for Round 1, if ≥75% of experts rated an item as ‘important but not critical’ or ‘critically important’ (scores 1–3 or 4–6), the item was retained for the next round. Items were removed if ≥15% of participants scored the item as ‘not important’ (scores 1–3).

For Round 2, items were only retained if ≥75% of experts rated an item as ‘critically important’ (scores 7–9) and if ≤15% of participants scored the item as ‘not important’ (scores 1–3).

In Round 1, any ‘could not rate’ answers were excluded from the analysis^79^; in Round 2, this option was not provided to ensure that the final consensus ratings reflected the views of all experts.

In Round 1 only, qualitative feedback was content‐analysed for important themes regarding phrasing, classification, duplication and suggestions for new items.

### 2.5 Ethics

Ethical approval for this study was granted by the University of (redacted for blind review) Department of Psychology Research Ethics Committee (code 18‐334).

## 3 RESULTS

### 3.1 Item generation

#### 3.1.1 Key informant interviews

Thematic analysis identified nine themes: Understanding, Listening and Communication, Taking Action, Practical Things, Empathy, Relationship, Staff Self‐compassion, Continuity of Care and Quality of Care. Subthemes within each of these themes were used to form the wording of potential items, incorporating participants' quotations as appropriate. This process generated a list of 48 candidate items (see Supporting Information S1: Tables E and F). Participants talked both about what compassionate care is and what it is not, resulting in both positively and negatively phrased items.

#### 3.1.2 Literature review

Results from the literature review of existing measures were integrated with the initial list of items and themes. This mapping exercise added nine items (see the Supporting Information material) to the themes of Understanding, Listening and Communication, Taking Action, Practical Things and Empathy.

#### 3.1.3 Revisions

**Figure 1:**
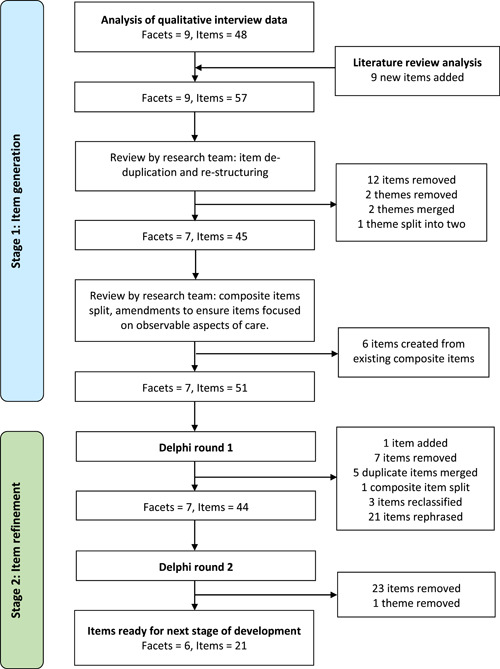
Flowchart of stages of measure development with structural changes.

There were two rounds of item revisions (see Figure 1) including removal of duplicates and compound items and restructuring themes. Item wording was amended to clarify focus on observable aspects of care. For example, ‘Feeling that staff understood what mattered most to me’ became ‘The things that mattered most to me were understood’. All items were reviewed for readability using the Flesch scales^80^ and rephrased into plain English with a first‐person, past tense voice.

A final list of 51 candidate items was organised within seven themes or conceptual facets of compassionate care: Understanding, Communication, Attention, Action, Emotional Sensitivity, Connection and Staff Self‐Compassion.

### 3.2 Item refinement using modified Delphi method

**Figure 2:**
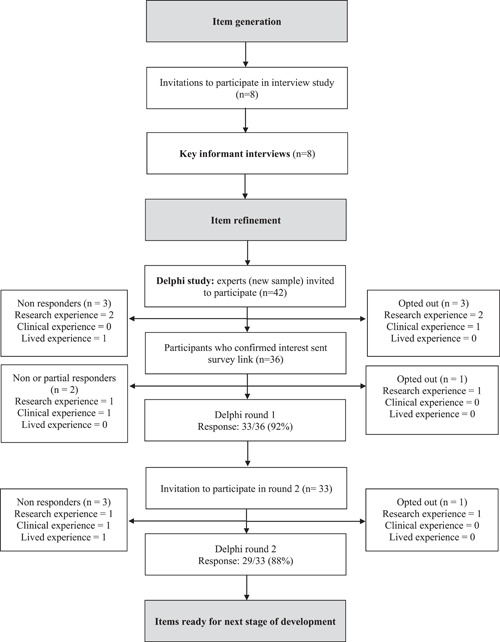
Flowchart of stages of measure development with number of responses.

Of the 42 individuals invited to the online Delphi survey, 36 confirmed and received the survey link for Round 1 (Figure 2) and 33 completed the survey (a Round 1 response rate of 92%). Three surveys were incomplete and were excluded from analysis. For Round 2, 33 people were invited and 29 completed the full survey (a Round 2 response rate of 88%).

#### 3.2.1 Round 1

Following Round 1, consensus was reached on retaining all 51 items (100%) for the next round at the predetermined level of either ‘important but not critical’ (4–6) or ‘critically important’ (7–9). No consensus was reached on any items rated as ‘not important’ (1–3); therefore, no items were removed on the basis of these ratings.

However, analysis of the qualitative feedback strongly indicated the removal and amendment of a number of items. This included removing the six negatively phrased items (e.g., ‘My problems were not acknowledged’) and one item that overlapped with the NHS Friends and Family Test.^81^ In addition, one new item was proposed and included (‘The staff member checked that I had understood what they said’), five duplicates were identified and merged into three items, one composite item was split into two and three items were reclassified under another facet. Twenty‐one items were slightly rephrased to improve clarity, and the facet ‘Emotional Capacity’ was renamed ‘Emotional Sensitivity’. This resulted in a final list of 44 items for the second round.

#### 3.2.2 Round 2

Round 2 led to consensus on 21 items (48%) rated as ‘critically important’ (7–9). Consensus was not reached on the remaining 23 items. Neither item in the ‘staff self‐compassion’ facet reached consensus and qualitative feedback indicated that patients would struggle to rate this item, so these items were excluded (see Supporting Information S1: Table G and ‘additional description of revisions’ in the Supporting Information material). Analysis of the qualitative feedback indicated that no additional classification or phrasing changes were required.

#### 3.2.3 Refined items

The item generation and refinement processes resulted in a list of potential measure items (see Supporting Information S1: Table H) consisting of 21 items organised into six facets: Understanding, Communication, Attention, Action, Emotional Sensitivity and Connection. These items are those that will be taken forward to the next phase of measure development.

## 4 DISCUSSION

This paper describes the systematic item development stages of a forthcoming measure of compassionate healthcare in action. These initial stages form the foundation for further testing and validation that we hope will ultimately result in a new patient‐reported measure of compassionate healthcare that focusses on the observable behaviours of compassionate care provided by a healthcare professional working in any clinical setting. This foundational work sought to be conceptually coherent, follow a rigorous development process^82^ and include PLE at all stages.

Initial development stages combined an inductive approach, by generating new questionnaire items from semi‐structured interviews, with a deductive approach, by gathering additional items from a literature review of existing measures. The measure development process gave prominence to what matters most to PLE of healthcare delivery at every stage. Items and facets were then refined through a two‐round modified online Delphi process with patient, clinician and research experts in compassionate care. Consensus was reached on 21 items including six facets: understanding, communication, attention, action, emotional sensitivity and connection. These items map onto both the affective and motivational components of compassion,^28^ indicating that the initial measure has conceptual coherence.^83^

Further scale development work on the format of the measure and face validity of these foundational items, with subsequent testing with a large community sample to establish, reliability, validity and factor structure, is underway.

### 4.1 Study strengths

The item development work for our proposed new measure has sought to focus on the following three things: (1) observable aspects of compassionate care delivery; (2) care in both physical health and mental health settings; and (3) a PLE involvement strategy throughout the initial phases. These are elements that we believe have not been realised by any one existing patient‐reported measure of compassionate care to date.

The prioritising of observable aspects of compassionate care complements recent empirical data that have started to map out the range of subtle verbal and nonverbal actions of healthcare staff.^38^, ^44^ By creating a list of items that aim to capture the specific behavioural components of a compassionate healthcare encounter,^42^ these items serve as a potential counterpoint to many items from existing measures which tend to use more subjective ‘I feel’ statements.^49^

The broad inclusion of patient and clinician perspectives from a range of different (physical and mental) healthcare contexts is different to the majority of questionnaire development papers in this field, where acute and primary care settings are more greatly represented (Table 1). Including mental health perspectives and perspectives relating to care given by a range of different roles may have allowed previously omitted aspects of care to be included, and may explain why some of our candidate items are not represented in any existing measures (e.g., the item relating to the staff member ‘doing what they said they would do’).

It remains to be seen whether this more inclusive approach, coupled with the prioritisation of greater PLE involvement at all stages alongside the use of formal consensus methods, will lead to stronger concept validity, specificity and practical applicability in a new measure of compassionate healthcare in action.

### 4.2 Potential clinical applications

This study represents the initial work for a measure of compassionate healthcare in action. Next steps in measure development are underway, and involve cognitive interviews to assess face validity, and establishment of reliability and validity using appropriate quantitative methods. In time, it is hoped that the resulting measure will be of use to clinical services in their measurement and improvement of compassionate healthcare. A measure of compassionate healthcare in action could be of particular use to services and clinicians wishing to monitor compassionate care provision and elicit meaningful feedback of use to staff training. A measure that can be used in both physical and mental healthcare settings could also be of use for comparisons across services. Any resulting final measure will be carefully framed in order to encourage interpretation of results from individual clinicians or services within the broader context of complex environmental and systemic factors that are known to influence the capacity of staff to provide compassionate care^10^, ^14^ and to discourage any reductionist approach to human compassion in clinical settings.

### 4.3 Limitations

Despite the research team's efforts to recruit a sample of patients and clinicians with a diverse range of healthcare experiences, the key informant and Delphi study samples were both largely homogeneous samples. It is possible that a larger, more diverse sample would have generated a different conceptualisation of observable compassionate care behaviours. For example, it is conceivable that an encounter with a healthcare professional is experienced very differently by patients who live in socioeconomically deprived areas with unequal access to health services compared to those who live in more affluent areas, and observable behaviours related to compassion may vary considerably across cultures. We hope to test the validity and acceptability of these initial items across different socioeconomic and cultural groups in subsequent phases of measure development.

The Delphi process was limited to two rounds and given the extensive changes to the structure and content of the measure between Rounds 1 and 2, a third round may have confirmed consensus and improved concurrent validity.^68^ Also, the Delphi participants did not receive an individualised report tracking their responses between rounds and in comparison with the group results,^78^ due to software limitations and the challenges of tracking changes following extensive restructuring between rounds. Researchers would benefit from the development of commonly agreed standards for the reporting of Delphi studies, particularly when used within measure development.

More broadly, it is important to note that at this stage of the development process, some items remain that relate more to felt states than observable behaviour (e.g., ‘I trusted the staff member’, ‘The staff member made me feel safe’ and ‘The things that matter most to me were understood’). These items may reflect an important interrelationship between the experiences of compassionate care, trust and safety described by interviewed participants. They may be indicative of a difficulty in separating out purely behavioural items for the construct of compassion. Their inclusion at this point in the measure development process reflects the inclusive approach used in this project (whereby all suggested items were put forward for review by the Delphi panel). However, they remain more subjective than objective in nature, and less in line with the core aims of the measure. In the next stage of scale development, cognitive interviews will be used to assess the face validity of these items in relation to the measurement of compassionate healthcare in action, and it remains to be seen if these ‘internal state’ items will be retained throughout this stage and the subsequent factor analytic stages of the project.

## 5 CONCLUSION

This paper presents the item development of a tool that aims to systematically measure what patients report are the observable behaviours of compassionate care delivery by a healthcare professional working in any healthcare setting. The study is both experientially and theoretically based, and PLE of healthcare delivery (patients and staff) were involved at all stages of its development. It combines inductive and deductive approaches and the use of a formal consensus method (modified online Delphi process) to produce an initial 21 items relating to six facets, ready for the next stage of exploration of reliability and validity, with the ultimate aim of publication of a final measure. We report on these initial stages in detail in the hope of encouraging greater transparency and replicability in measure development and to emphasise the value of PLE involvement across all stages of any new patient‐reported measure.

## AUTHOR CONTRIBUTIONS

**Eleanor Chatburn**: Conceptualisation; investigation; methodology; project administration; resources; writing—original draft; writing—review and editing. **Elizabeth Marks**: Conceptualisation; investigation; methodology; supervision; writing—review and editing. **Lucy Maddox**: Conceptualisation; investigation; methodology; supervision; writing—review and editing.

## CONFLICT OF INTEREST STATEMENT

The authors declare no conflicts of interest.

## Supporting information

Supporting information.

## Data Availability

Data are available on request from the authors.
