# Supplementary material for: Item development for a patient‐reported measure of compassionate healthcare in action
Source: Health Expect. 2024 Jan 21;27(1):e13953. doi: 10.1111/hex.13953 (PMC10801284; doi:10.1111/hex.13953)
Supplement: Supplementary file 1 — Supporting information. [file HEX-27-e13953-s001.docx]

**Supplementary material**

**Table A**

*Categorisation of the Content of Existing Measures*

Note. Items are rated as either: S subjective, B behavioural, or U unclear.

***Burnell & Agan, 2013: Compassionate Care Assessment Tool:***

| 1. Having a sense of humor 2. Providing unconditional love/respect 3. Supporting spiritual beliefs 4. Providing access to spiritual support 5. Excusing shortcomings 6. Possessing inner beauty 7. Providing outside connection 8. Dealing with difficult issues. 9. Controlling pain 10. Giving timely treatments 11. Checking frequently 12. Including in plan of care 13. Presenting professional image. 14. Encouraging 15. Appreciating patient and family 16. Considering of personal needs 17. Being empathetic. 18. Appearing competent 19. Displaying confidence 20. Showing skill (with equipment). | 1. S 2. U 3. S 4. B 5. S 6. S 7. S 8. B 9. B 10. B 11. B 12. B 13. S 14. S 15. S 16. S 17. S 18. S 19. S 20. B |
| --- | --- |
| **Total:** | **B = 7**  **S = 12**  **U = 1**  **Majority subjective** |

***Fogarty: [Unnamed physician compassion scale]:***

| 1. Wants what is best for the patient vs wants what is best for himself. 2. Cares about the patient vs does not care about the patient. 3. Acknowledges patient’s emotions vs ignores patient's emotions 4. Encourages patient’s questions vs discourages patient's questions 5. Encourages patient involvement in treatment decision vs discourages patient involvement in treatment decision | 1. S 2. S 3. B 4. B 5. B |
| --- | --- |
| Total: | **B = 3**  **S = 2**  **U = 0**  **Roughly equal** |

***Lown et al., 2015:*** ***Schwartz Center Compassionate Care Scale:***

| 1. Express sensitivity, caring and compassion for your situation? 2. Strive to understand your emotional needs? 3. Consider the effect of your illness on you, your family, and the people most important to you? 4. Listen attentively to you? 5. Convey information to you in a way that was understandable? 6. Gain your trust? 7. Always involve you in decisions about your treatment? 8. Comfortably discuss sensitive, emotional, or psychological issues? 9. Treat you as a person not just a disease? 10. Show respect for you, your family and those important to you? 11. Communicate test results in a timely and sensitive manner? 12. Spend enough time with you? | 1. S 2. U 3. B 4. B 5. B 6. S 7. B 8. B 9. S 10. B 11. B 12. B |
| --- | --- |
| Total: | **B = 8**  **S = 3**  **U = 1**  **Majority behavioural** |

***Mercer et al., 2004: Consultation And Relational Empathy Measure:***

| 1. Making you feel at ease ((introducing him/herself, explaining his/her position, being friendly and warm towards you, treating you with respect; not cold or abrupt)) 2. Letting you tell your story (giving you time to fully describe your condition in your own words; not interrupting, rushing or diverting you) 3. Really listening (paying close attention to what you were saying; not looking at the notes or computer as you were talking) 4. Being interested in you as a whole person (asking/knowing relevant details about your life, your situation; not treating you as "just a number") 5. Fully understanding your concerns (communicating that he/she had accurately understood your concerns and anxieties; not overlooking or dismissing anything ) 6. Showing care and compassion (seeming genuinely concerned, connecting with you on a human level; not being indifferent or "detached") 7. Being positive (having a positive approach and a positive attitude; being honest but not negative about your problems) 8. Explaining things clearly (fully answering your questions; explaining clearly, giving you adequate information; not being vague) 9. Helping you to take control (exploring with you what you can do to improve your health yourself; encouraging rather than "lecturing" you) 10. Making a plan of action with you (discussing the options, involving you in decisions as much as you want to be involved; not ignoring your views) | 1. U 2. B 3. B 4. B 5. B 6. S 7. U 8. B 9. B 10. B |
| --- | --- |
| Total: | **B = 7**  **S = 1**  **U = 2**  **Majority behavioural** |

***Roberts et al., 2019: 5-Item Tool to Measure Patient Assessment of Clinician Compassion:***

| 1. How often do you feel your provider cares about your emotional or psychological well-being? 2. How often do you feel your provider is interested in you as a whole person? 3. How often do you feel your provider is considerate of your personal needs? 4. How often do you feel your provider is able to gain your trust? 5. How often do you feel your provider shows you care and compassion? | 1. S 2. S 3. S 4. S 5. S |
| --- | --- |
| Total: | **B = 0**  **S = 5**  **Subjective** |

***Sinclair: Sinclair Compassion Questionnaire (Long-Form)***

| 1. My Healthcare Providers made me feel cared for. 2. My Healthcare Providers showed genuine concern for me. 3. My Healthcare Providers communicated with me in a sensitive manner. 4. I felt that my Healthcare Providers were attentive to me. 5. My Healthcare Providers provided me with comfort. 6. My Healthcare Providers were very supportive when they talked with me. 7. My Healthcare Providers provided care in a gentle manner. 8. My Healthcare Providers spoke to me with kindness 9. My Healthcare Providers saw me as a person and not just as a patient. 10. My Healthcare Providers behaved in a caring way. 11. My Healthcare Providers really understood my needs. 12. I had a good relationship with my Healthcare Providers. 13. My Healthcare Providers were able to see things from my perspective 14. My Healthcare Providers had a warm presence. 15. I felt that my Healthcare Providers were sincere. | 1. S 2. B 3. B 4. S 5. U 6. B 7. S 8. B 9. U 10. S 11. S 12. S 13. S 14. S 15. S |
| --- | --- |
| Total: | **B = 4**  **S = 9**  **U = 2**  **Majority subjective** |

## Table B

*Demographics of Participants in Key Informant Interviews (n=8).*

| Age (years) | Gender | Ethnicity | Location | Role | Area of health expertise | Specific areas of professional/lived experience |
| --- | --- | --- | --- | --- | --- | --- |
| 43 | F | White British | UK: South east | General practitioner | Physical health | General practice; Emergency medicine |
| 49 | M | Indian | UK: South east | Medical consultant | Physical health | Audio-vestibular medicine |
| 39 | F | White British | UK: South west | Clinical psychologist | Mental health | Older adult mental health; geriatrics |
| 60 | M | White British | UK: South west | Patient/service user | Mental health | A&E; Psychiatric liaison services |
| 29 | F | White British | UK: Midlands | Patient/service user | Physical health | Obstetrics and maternity |
| 46 | F | White British | UK: South west | Patient/service user | Mental health | Inpatient mental healthcare |
| 56 | F | White British | UK: South east | Nurse manager | Mental health | Inpatient mental healthcare |
| 44 | M | White European | UK: North east | Patient/service user | Physical health | Neurodegenerative medicine |

## Table C

*Interview Schedule for Key Informant Interviews*

| Questions asked of all interviewees (clinicians and patients) | Additional questions asked of clinicians |
| --- | --- |
| Q1 Could you describe in your own words what “compassionate care” means to you?  *Example prompts:*  When I say the word “compassion”, what comes to mind?  Q2 Could you describe a specific time when you experienced compassionate care from a healthcare professional, team, or service?  *Example prompts:*  If I were a fly on the wall in that room, what would I have seen the staff member doing or not doing?  How did that make you/your family feel?  Q3 Could you describe a specific time when you did not experience compassionate care from a healthcare professional, team, or service?  *Example prompts:*  If I were a fly on the wall in that room, what would I have seen the staff member doing or not doing?  Q4 Based on your knowledge and expertise, what else is important for us to consider when defining compassionate care?  *Example prompts:*  Is there anything I have not asked you that you think is important for me to know? | Q5 Could you describe a specific time when you feel that you successfully delivered compassionate care?  *Example prompts*:  What would I have seen you doing or not doing in the room?  What do you think enabled you to do that?  How did it make you feel?  How do you think it made your patient/their family feel?  Q6 Could you describe a specific time when you felt that you were less successful in delivering compassionate care?  *Example prompts:*  What would I have seen you doing or not doing in the room?  What do you think got in the way of you delivering compassionate care in that moment?  How did it make you feel?  How do you think it made your patient/their family feel? |

## Table D

*Demographics of Participants in the Modified Online Delphi Process*

|  | | Round 1  n (%) | Round 2  n (%) |
| --- | --- | --- | --- |
| **All participants** | | 32 | 29 |
| **Gender (self-identified)** | | | |
| Female | | 22 (66%) | 19 (65%) |
| Male | | 11 (33%) | 10 (35%) |
| **Ethnicity (self-identified)** | | |  |
| White British | | 27 (84%) | 24 (83%) |
| All other types | | 5 (17%) | 5 (17%) |
| **Age (years)** | | |  |
| 25-34 | | 5 (15%) | 5 (17%) |
| 35-44 | | 12 (37%) | 10 (34%) |
| 45-54 | | 6 (19%) | 5 (17%) |
| 55-64 | | 7 (21%) | 7 (24%) |
| 65+ | | 3 (10%) | 3 (10%) |
| **Location** | |  |  |
| South East | | 6 (19%) | 6 (21%) |
| South West | | 9 (28%) | 7 (24%) |
| London | | 3 (10%) | 3 (10%) |
| East | | 2 (6%) | 2 (7%) |
| Midlands | | 4 (13%) | 4 (14%) |
| North East | | 2 (6%) | 1 (3%) |
| Scotland | | 2 (6%) | 2 (7%) |
| UK - not specified | | 3 (10%) | 3 (10%) |
| International | | 2 (6%) | 2 (6%) |
| **Expert type and recruitment channel*** | | |  |
| Lived experience (total), of which:  PLE committee or personal contact  Twitter | 13 (41%)  7  6 | | 12 (41%)  7  5 |
| Researchers – personal contacts/internet research | 5 (16%) | | 5 (17%) |
| Clinicians* – personal contacts/internet research | 14 (43%) | | 12 (41%) |
| **Self-reported nature of expertise***(NB option to select more than one)* | | |  |
| Significant personal experience in and experience of receiving compassionate care | | 19 (59%) | 18 (62%) |
| Significant research interest in compassion or in delivering compassionate care | | 21 (65%) | 20 (69%) |
| Significant clinical practice in field of compassion or delivering compassionate care | | 15 (47%) | 14 (48%) |
| **Area of healthcare expertise** *(NB option to select more than one)* | | |  |
| Mental health | | 29 (90%) | 26 (90%) |
| Physical health | | 19 (60%) | 19 (66%) |
| Social care | | 7 (22%) | 6 (21%) |
| All aspects of healthcare | | 7 (22%) | 6 (21%) |
| Other – inc educational settings, substance use | | 4 (13%) | 3 (10%) |

Note: The ‘clinician’ category includes anyone who has professional training in delivering healthcare; some of these participants also had significant research expertise in compassionate care but they are not counted twice. Any low frequency group characteristics have been obscured.

## Table E

*Themes and Items Generated from Qualitative Interview Data and Literature Review (LR) (Before Process of Initial Item Refinement)*

| Theme | Total items | Items from interviews | Additional items from LR | Source/s and original item wording |
| --- | --- | --- | --- | --- |
| Understanding | 7 | 5 | 2 | “Staff are interested in you as a whole person” (CARE).  “Staff strive to understand your emotional needs” (SCCCS). |
| Taking Action. | 8 | 7 | 1 | “Always involve you in decisions about your treatment?” (SCCCS). |
| Practical Things | 10 | 8 | 2 | “Did staff spend enough time with you?” (SCCCS).  “Staff are positive” (CARE). |
| Listening & Communication | 15 | 12 | 3 | “Staff let you tell your story” (CARE)  “Communicate information in a timely and sensitive manner?” (SCCCS).  “Giving timely treatments” (CCAT). |
| Empathy | 4 | 3 | 1 | “Comfortably discuss sensitive, emotional or psychological issues?” (SCCCS). |
| Relationship. | 5 | 5 | 0 | n/a |
| Staff Self-compassion. | 3 | 3 | 0 | n/a |
| Continuity of Care | 4 | 4 | 0 | n/a |
| Quality of Care | 1 | 1 | 0 | n/a |
| TOTALS | 58 | 48 | 9 |  |

*Note.* Abbreviations used in table: CCAT (Burnell & Agan, 2013), CARE (Mercer et al., 2004), SCCCS (Lown et al., 2015).

## Table F

*Themes and Items Generated from Key Informant Interviews*

| Theme | Sub-themes | Interviewee identifier |
| --- | --- | --- |
| Understanding (5) | Staff show they understand the context and impact of the problem from your perspective (they have an “holistic understanding”). | 1,2,3,4 |
|  | Feeling that staff understand “what matters most” to you (i.e. values) | 2,4,7 |
|  | (N) Feeling like you are “not listened to” and your problems are “not acknowledged”. | 2,5 |
|  | Having your problems and distress “taken seriously” (and not ignored or dismissed). | 2,3,4 |
|  | Feeling that someone makes time for you, understands you, “genuinely cares”, and wants to help you. | 3,4,5,6 |
| Listening & communication (12) | Staff “listen actively” and then check their understanding with you | 1,2,3,5 |
|  | Staff validate your experience/distress. | 1,3 |
|  | Staff help to externalise the problem (e.g. “that isn’t you, that’s not right”). | 4 |
|  | (N) Staff do not impose their own agenda and “do not judge” you. | 1,2,3,5 |
|  | Staff “personalise” their communication style to suit you. | 4,8 |
|  | Staff take time to explain in plain language what is happening to you and what they are going to do. | 2,3,4,5 |
|  | Staff give you a “straight answer” to your questions. | 4,8 |
|  | (N) Staff do not give you the opportunity to ask questions leaving you feeling uncertain or confused. | 1,4,5 |
|  | The clinician says hello and introduces themselves. | 1,3,8 |
|  | The clinician uses “gentle”, “non-threatening” tone of voice and an open body posture. | 3,5,7,8 |
|  | (N) Staff did not raise their voice or shout at you. | 5,7,8 |
|  | Staff are polite and “respectful” at all times (e.g. even if tired, busy). | 5,8 |
| Taking action (7) | Feeling that someone “genuinely cares” about you and wants to help. | 3,4,5,6 |
|  | Feeling that staff/services are doing everything possible to help you. | 2,3,5,8 |
|  | Feeling that staff/services “want the best” for you (acting in best interests/ advocating for you. | 4,6 |
|  | “Knowing that services can provide you with a safe and secure place” (where you will get help). | 3,4,6,7 |
|  | Staff help you to “make a plan”. | 1,5,8 |
|  | Staff help you so that you can do things for yourself. | 1,5,8 |
|  | Sensing that staff “see a way through the problem” (i.e. they know how to help you). | 1,4,6 |
| Practical things (8) | Staff “chat” with you and help you to feel at ease. | 3,5,6,7 |
|  | Staff “go the extra mile” to make you feel comfortable. | 3,5,6,7 |
|  | Staff make appropriate use of touch so that you feel there is a human “connection”. | 4,5,7 |
|  | When talking to you, the clinician faced you, made eye contact, and gave you their “full attention”. | 3,5 |
|  | (N) Staff not appearing rushed, distracted, and that they do not have time for you. | 5 |
|  | (N) Staff ignored, dismissed, or delayed responding to your requests for help. | 2,4 |
|  | (N) Staff appeared “cold, rude, distant”, dismissive, or uninterested towards you or your family. | 1,3,4,5,6,7 |
|  | Staff follow through on their promises (i.e. do what they say they will do). | 1,6,7,8 |
| Empathy (3) | Staff show they are able to see things from your perspective. | 7,8 |
|  | Staff empathise with you and your experience or problem. | 1,2,3,5,6 |
|  | Sensing that you and the staff have a shared “emotional connection” . | 5,6,7 |
| Relationship (5) | Feeling you are “able to trust” staff with your care. | 1,6,7,8 |
|  | Feeling that you have formed a good connection with staff (“we just clicked”). | 1,3,4,6,7 |
|  | Feeling like you and staff are “all on the same team”. | 6 |
|  | Feeling that you are treated as a human being (and not as an object, number, or diagnosis). | 1,3,4,6,7 |
|  | Having a sense of a “shared humanity”. | 6,7 |
| Staff Self-compassion (3) | The staff member “knows how to be compassionate to themself” (self-compassion). | 3,4 |
|  | You sense that staff have “emotional maturity” and self-awareness. | 4,7 |
|  | You sense that staff look after their own self-care. | 3,4 |
| Continuity of Care (4) | Services/staff understand the patient’s own journey through the healthcare system. | 2,5 |
|  | Services/staff think about the whole patient experience (from referral to discharge). | 1,2,5 |
|  | Staff show they “know what the patient knows”. | 1,3,4,5 |
|  | There is no communication breakdown between staff members/teams/services. | 1,3,5 |
| Care Quality (1) | Sensing that staff “would treat their own loved ones this way”. | 6 |

*Note.* Includes illustrative quotes and interviewee identifier (interviewees numbered 1-8). (N) = negatively phrased items.

## Table G

*Changes in Number of Items and Facets Before and During the Delphi Process*

| Facets | Number of items in each facet | | |
| --- | --- | --- | --- |
|  | Pre-Delphi | Post-round 1 Delphi | Post-round 2 Delphi |
| Understanding | 6 | 4 | 2 |
| Attention | 6 | 5 | 2 |
| Communication | 8 | 7 | 4 |
| Action | 16 | 12 | 4 |
| Emotional sensitivity | 6 | 7 | 5 |
| Connection | 7 | 7 | 4 |
| Staff self-compassion | 2 | 2 | 0 |
| **Totals** | **51** | **44** | **21** |

## Table H

*Results of Delphi Round Two Survey*

| **Theme** | **Example items** |
| --- | --- |
| Understanding | I was taken seriously. |
| Attention | The staff member made time for me. |
| Communication | The staff member explained clearly what was happening to me and what would happen next. |
| Action | The staff member did what they said they would do. |
| Emotional sensitivity | The staff member was comfortable discussing sensitive issues with me. |
| Connection | The staff member treated me with respect. |

## Completed COREQ (Consolidated Criteria for Reporting Qualitative Research 31-Item Checklist) for key informant interview component of the study

**Domain 1: Research team and reflexivity**

Personal Characteristics

1. Interviewer/facilitator: Which author/s conducted the interview or focus group?

*The main researcher (EC) conducted all the key informant interviews.*

2. Credentials: What were the researcher’s credentials? E.g. PhD, MD

*M.A. for main researcher, DClinPsy for both supervisors.*

3. Occupation: What was their occupation at the time of the study?

*EC was a Doctorate in Clinical Psychology student at University of Bath. EM and LM are both clinical psychologists. EM was a lecturer at University of Bath and LM was Senior Clinical Advisor for the British Association of Behavioural and Cognitive Psychotherapies.*

4. Gender: Was the researcher male or female?

*All three members of the research team identify as female.*

5. Experience and training: What experience or training did the researcher have?

*The main researcher has experience with qualitative research and at the time of writing was completing three years of doctoral level training in clinical psychology.*

Relationship with participants

6. Relationship established: Was a relationship established prior to study commencement?

*All participants were recruited via professional contacts so were previously known to one member of the research team. The interviewer previously knew four interviewees: three in a professional capacity and one in a personal capacity. For all other participants, a relationship was established by email where the rationale for the study and participant role was explained.*

7. Participant knowledge of the interviewer: What did the participants know about the researcher? e.g. personal goals, reasons for doing the research.

*The interviewer introduced herself and reasons for doing the study via email and at the beginning of the interview.*

8. Interviewer characteristics: What characteristics were reported about the interviewer/facilitator? e.g. Bias, assumptions, reasons and interests in the research topic.

*The interviewer was interested in the topic following discussions within the research team about the lack of behaviourally based patient reported measures in this area. As clinicians (trainee clinician in the case of the main researchers), all members of the research team had lived experience both of receiving and providing compassionate healthcare.*

**Domain 2: study design**

Theoretical framework

9. Methodological orientation and Theory: What methodological orientation was stated to underpin the study? e.g. grounded theory, discourse analysis, ethnography, phenomenology, content analysis

*No specific theoretical framework underpinned the analysis.*

Participant selection

10. Sampling: How were participants selected? e.g. purposive, convenience, consecutive, snowball.

*Purposive sampling.*

11. Method of approach: How were participants approached? e.g. face-to-face, telephone, mail, email.

*All participants were approached via email for the interview study.*

12. Sample size: How many participants were in the study?

*In total 8 interviews were conducted, 4 with clinicians and 4 with people with lived experience of receiving compassionate care. All the clinicians also identified with having lived experience of receiving compassionate care either as a patient or carer/relative.*

13. Non-participation: How many people refused to participate or dropped out? Reasons?

*No participants withdrew from the interview study.*

Setting

14. Setting of data collection: Where was the data collected? e.g. home, clinic, workplace.

*The interviews were primarily conducted via telephone. Two interviews were conducted face to face in a university building.*

15. Presence of non-participants Was anyone else present besides the participants and researchers?

*No one else was present during the interviews.*

16. Description of sample: What are the important characteristics of the sample? e.g. demographic data, date.

*Demographic characteristics are reported in detail in the main paper.*

Data collection

17. Interview guide: Were questions, prompts, guides provided by the authors?

*The interview guide is reported in detailed in the supplementary table.*

18. Repeat interviews: Were repeat interviews carried out? If yes, how many?

*No repeat interviews were conducted.*

19. Audio/visual recording: Did the research use audio or visual recording to collect the data?

*Interviews were audio recorded with the consent of participants.*

20. Field notes: Were field notes made during and/or after the interview or focus group?

*The interviewer made brief field notes immediately after each interview.*

21. Duration: What was the duration of the interviews or focus group?

*Interviews lasted from 15 to 35 minutes in duration.*

22. Data saturation: Was data saturation discussed?

*Data saturation was discussed in the research team. Guidelines were followed from Braun and Clark about appropriate sample size for a project of this nature.*

23. Transcripts returned: Were transcripts returned to participants for comment and/or correction?

*Transcripts were not returned to participants.*

**Domain 3: analysis and findings**

Data analysis

24. Number of data coders: How many data coders coded the data?

*See manuscript for full description.*

25. Description of the coding tree: Did authors provide a description of the coding tree?

*See manuscript for full description.*

26. Derivation of themes: Were themes identified in advance or derived from the data?

*See manuscript for full description.*

27. Software: What software, if applicable, was used to manage the data?

Interviews were coded in MS Word.

28. Participant checking: Did participants provide feedback on the findings?

*Due to time constraints, participants were not given the opportunity to provide feedback*.

Reporting

29. Quotations presented: Were participant quotations presented to illustrate the themes / findings? Was each quotation identified? e.g. participant number.

*The themes in the results section of the manuscript are illustrated using quotations and each quotation includes a participant number.*

30. Data and findings consistent: Was there consistency between the data presented and the findings?

*As a research team we believe the data and findings are consistent.*

31. Clarity of major themes: Were major themes clearly presented in the findings?

*We have aimed to present the major themes clearly in the results section, given the context that this interview study was one part of a larger study and there was space constraints within an article of this length.*

32. Clarity of minor themes: Were minor themes clearly presented in the findings?

*We have aimed to present the minor themes clearly in the results section, given the context that this interview study was one part of a larger study and there was space constraints within an article of this length.*

## Additional description of revisions conducted by research team following item generation process

In round one, the “Taking Action” and “Practical Things” themes were merged into a new “Action” theme. “Listening and Communication” was split into two themes: “Attention” and “Communication”. The “Empathy” theme was renamed “Emotional capacity” and “Relationship” renamed “Connection”. The research team judged that the “Continuity of Care” and “Quality of Care” themes both represented different constructs to compassionate care; these themes were removed but the items were retained. Items from the “Continuity of Care” theme were moved to “Action” and “Communication” and the single item labelled as “Quality of Care” was moved to “Connection”. A process of de-duplication led to removal of 12 items in total. For example, “staff understand the problem from your perspective” (source: interview) was merged with “staff are interested in you as a whole person” (source: CARE Measure) to create “the staff member took the time to understand all of my concerns”.

A second round of revisions identified a number of compound items containing multiple concepts e.g. “They did what they said they would do and stuck to their promises”. Therefore, all compound items were split, creating six new items. The researchers also amended item wording to ensure a clear focus on observable aspects of care. For example, “feeling that staff understood what mattered most to me” was reworded as “the things that mattered most to me were understood”.

Finally, all items were reviewed carefully for readability using the Flesch scales (Flesch, 1948) and were rephrased into plain English using a consistent first-person, past tense voice. For example, the original item “Staff emphasise with you and your problem” was revised to “The staff member showed that they could see things from my perspective”.
